# Contribution of Functional Divergence Through Copy Number Variations to the Inter-Species and Intra-Species Diversity in Specialized Metabolites

**DOI:** 10.3389/fpls.2019.01567

**Published:** 2019-11-26

**Authors:** Kazumasa Shirai, Kousuke Hanada

**Affiliations:** Department of Bioscience and Bioinformatics, Kyushu Institute of Technology, Fukuoka, Japan

**Keywords:** specialized metabolite, adaptation, *Arabidopsis*, copy number variant, gene duplication

## Abstract

There is considerable diversity in the specialized metabolites within a single plant species (intra-species) and among different plant species (inter-species). The functional divergence associated with gene duplications largely contributes to the inter-species diversity in the specialized metabolites, whereas the intra-species diversity is due to gene dosage changes *via* gene duplications [i.e., copy number variants (CNVs)] at the intra-species level of evolution. This is because CNVs are thought to undergo associated with less functional divergence at the intra-species level of evolution. However, functional divergence caused by CNVs may induce specialized metabolite diversity at the intra-species and inter-species levels of evolution. We herein discuss the functional divergence of CNVs in metabolic quantitative trait genes (mQTGs). We focused on 5,654 previously identified mQTGs in 270 *Arabidopsis thaliana* accessions. The ratio of nonsynonymous to synonymous variations tends to be higher for mQTGs with CNVs than for mQTGs without CNVs within *A. thaliana* accessions, suggesting that CNVs are responsible for the functional divergence among mQTGs at the intra-species level of evolution. To evaluate the contribution of CNVs to inter-species diversity, we calculated the ratio of nonsynonymous to synonymous substitutions in the *Arabidopsis* lineage. The ratio tends to be higher for the mQTGs with CNVs than for the mQTGs without CNVs. Additionally, we determined that mQTGs with CNVs are subject to positive selection in the *Arabidopsis* lineage. Our data suggest that CNVs are closely related to functional divergence contributing to adaptations *via* the production of diverse specialized metabolites at the intra-species and inter-species levels of evolution.

## Introduction

Plants produce various specialized metabolites, the diversity of which is closely related to adaptive evolution (Pichersky and Lewinsohn, 2011). Specialized metabolites vary among different species as well as within single species (Chan et al., 2010; Weigel, 2012; Carreno-Quintero et al., 2013; Alseekh et al., 2015; Matsuda et al., 2015). The diversity of the specialized metabolites resulted from gene duplications among various plant species. We previously revealed that copy number variants (CNVs) derived from gene duplications are associated with specialized metabolites (Shirai et al., 2017).

Gene duplications contribute to the diversity in specialized metabolites because of two possible effects. The first effect is functional divergence. After gene duplication events, the copied genes tend to accumulate nonsynonymous mutations because of relaxed selection pressures (Scannell and Wolfe, 2008). Consequently, the copied genes induce functional divergence (Ohno, 1970), ultimately leading to the variability in the specialized metabolites among various plant species (Hanada et al., 2008; Kliebenstein and Osbourn, 2012; Panchy et al., 2016). The second effect involves gene dosage changes. Specifically, gene duplications increase gene dosage (Ohno, 1970). In particular, CNVs are believed to be the main cause of intra-species gene dosage changes (Zmienko et al., 2014). There is experimental evidence that the abundance of specialized metabolites within a single species is critically controlled by altered gene dosages due to CNVs (Kliebenstein, 2001). However, it remains unclear whether CNVs associated with specialized metabolites tend to induce functional divergence at the genomic scale.

We herein discuss the functional divergence of CNVs associated with specialized metabolites. For this discussion, we performed additional analyses involving our previously published data. On the basis of the analyses, we propose that CNVs induce functional divergence that generates various specialized metabolites during the evolution of *A. thaliana*.

### Functional Divergence of CNVs at the Intra-Species Level of Evolution

It is believed that CNVs mainly cause quantitative changes rather than qualitative changes (Zmienko et al., 2014), likely because of an insufficient amount of time for CNVs to accumulate nonsynonymous mutations leading to the diversity in specialized metabolites. However, several studies have identified a few nonsynonymous mutations responsible for the functional divergence of genes related to specialized metabolites (Chye et al., 2000; Yu et al., 2015; Bunsupa et al., 2016). These reports suggest CNVs may induce functional divergence.

To examine the functional divergence of duplicated genes, the selection pressure based on the ratio between the nonsynonymous mutation/substitution rate and the synonymous mutation/substitution rate is useful (Hanada et al., 2009). High and low selection pressures are associated with functional divergence and constraint, respectively. Therefore, we estimated the dN_SNP_/dS_SNP_ ratio, which is the ratio between the number of nonsynonymous mutations relative to the number of nonsynonymous sites (dN_SNP_) and the number of synonymous mutations relative to the number of synonymous sites (dS_SNP_) (Nei and Gojoborit, 1986; Hanada et al., 2009), for 27,130 annotated protein-coding genes in 270 A. *thaliana* accessions.

The single nucleotide polymorphism (SNP) data examined in this study were compiled from 270 A. *thaliana* accessions analyzed in several studies (http://1001genomes.org, 1001 Genomes; Mouille et al., 2006; Cao et al., 2011; Gan et al., 2011; Schmitz et al., 2013; Shirai et al., 2017. A total of 7,624,270 SNPs were included. For each accession, nonsynonymous and synonymous variations were annotated according to the TAIR10 database with the SnpEff program (https://www.arabidopsis.org; Cingolani et al., 2012). Of the 7,624,270 SNPs, 1,330,920 were located in 27,130 annotated protein-coding genes in the reference *A. thaliana* genome. For each of the 270 accessions, the 1,330,920 SNPs were classified as 733,796 nonsynonymous and 597,124 synonymous mutations in the 27,130 coding sequences. There was an average of 27 nonsynonymous and 22 synonymous mutations in each of the 27,130 coding sequences. Because the number of nonsynonymous and synonymous sites in codons varied, we calculated the number of synonymous and nonsynonymous sites in all 27,130 coding sequences with scripts that we developed following the Nei-Gojobori method (Nei and Gojoborit, 1986; Hanada et al., 2009). The 27,130 genes were classified as metabolic quantitative trait genes (mQTGs) and mQTGs with CNVs. We previously predicted 5,654 mQTGs for 1,335 specialized metabolites in *A. thaliana* (Shirai et al., 2017). In that study, mQTGs were detected by combining a genome-wide association study (GWAS) and a metabolite-transcriptome correlation analysis (MTCA). This method enabled the prediction of mQTGs with a lower false positive rate than that of the general GWAS method. Genes with CNVs were previously detected by comparing genomic read counts among *A*. *thaliana* accessions (Gan et al., 2011). Of the 27,130 genes, 929 were predicted as genes with CNVs (*P* < 0.05).

**Figure 1:**
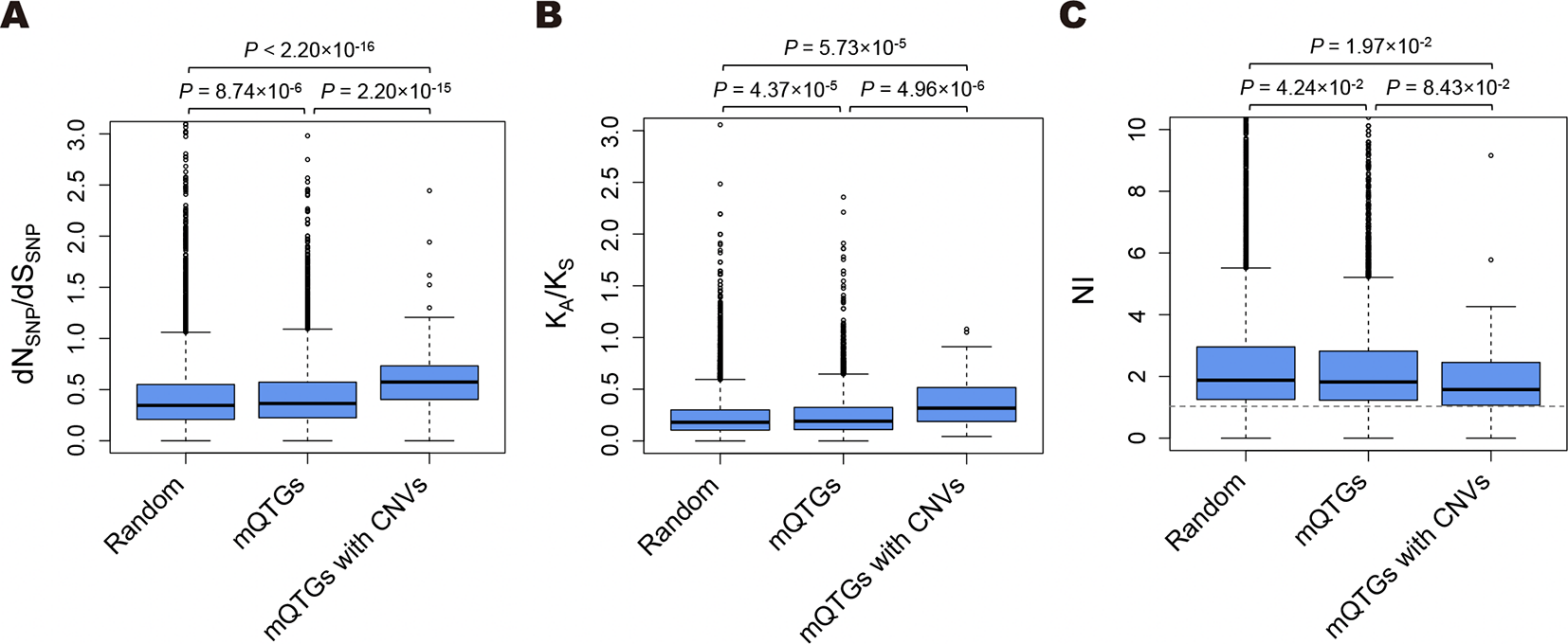
Comparison of the functional divergence and the selection pressure in *Arabidopsis*. **(A)** Box plots present the dN_SNP_/dS_SNP_ ratios of *Arabidopsis thaliana* accessions. **(B)** Box plots present the K_A_/K_S_ ratios between *A*. *thaliana* and *Arabidopsis lyrata*. **(C)** Box plots present the NIs between *A*. *thaliana* and *A*. *lyrata*. Random, 10,000 randomly selected genes; mQTGs, metabolic quantitative trait genes; mQTGs with CNVs, metabolic quantitative trait genes with copy number variants. In each box plot, the box represents the 25%–75% range, the middle line represents the median, the dotted line represents the 1%–99% range, and the outer circles are outliers. *P* values were calculated with the Wilcoxon rank sum test. The horizontal dotted line represents NI = 1.0.

To assess whether the functional divergence of CNVs is associated with the diversity in specialized metabolites, we compared the dN_SNP_/dS_SNP_ ratios among mQTGs, mQTGs with CNVs, and randomly selected genes (**Figure 1A** and **Supplementary Table S1**). The dN_SNP_/dS_SNP_ ratios were significantly higher for the mQTGs and mQTGs with CNVs than for the randomly selected genes (Wilcoxon rank sum test: *P* < 0.001; **Figure 1A**). Moreover, the ratios of mQTGs with CNVs were also significantly higher than the ratios of mQTGs (Wilcoxon rank sum test: *P* < 0.001; **Figure 1A**). These results imply that nonsynonymous variations tend to accumulate in mQTGs more frequently than in genes not associated with specialized metabolites. Specifically, mutations that alter the amino acid sequence accumulated in mQTGs with CNVs at a higher rate than in mQTGs without CNVs. These findings suggest that the diversity in specialized metabolites due to CNVs is the result of the functional divergence of mQTGs in addition to gene dosage changes at the genomic scale. Additionally, mQTGs with CNVs tended to be associated with a larger number of specialized metabolites than mQTGs without CNVs (Wilcoxon rank sum test: *P* = 1.92 × 10^−3^; **Supplementary Figure S1**), implying that the functional divergence derived from CNVs enhances the divergence of specialized metabolites.

It was unclear whether CNVs induce functional divergence for mQTGs only or for other genes as well. Therefore, we compared the dN_SNP_/dS_SNP_ ratios of randomly selected genes and non-mQTGs with CNVs. The dN_SNP_/dS_SNP_ ratios were significantly higher for the non-mQTGs with CNVs than for the randomly selected genes (Wilcoxon rank sum test: *P* < 2.2 × 10^−16^; **Supplementary Figure S2**). Thus, CNVs are generally responsible for the functional divergence of genes at the intra-species level of evolution.

### Functional Divergence of CNVs at the Inter-Species Level of Evolution

It was recently reported that CNVs are associated with various phenotypic differences within a plant species (Lye and Purugganan, 2019). By contrast, the contribution of CNVs to inter-species diversity remains relatively unknown in plants.

The dN_SNP_/dS_SNP_ ratio indicates the intra-species level of evolution. Therefore, to characterize the functional divergence of CNVs at the inter-species level of evolution, we examined the K_A_/K_S_ ratio, which is the ratio between the number of nonsynonymous substitutions relative to the number of nonsynonymous sites (K_A_) and the number of synonymous substitutions relative to the number of synonymous sites (K_S_). The K_A_/K_S_ ratio was estimated for 20,498 orthologs between *A*. *thaliana* and *Arabidopsis lyrata*. These orthologs were detected based on the reciprocal best hit (E-value < 1.0 × 10^−3^ and coverage > 90%) of a BLASTP (version 2.8.1) analysis of *A*. *thaliana* and *A*. *lyrata* (https://www.arabidopsis.org, TAIR10; http://genome.jgi.doe.gov, Phytozome v12: Alyrata_384_v2.1; Rawat et al., 2015; Boratyn et al., 2013). The coding sequences were aligned according to the amino acid sequences aligned by MAFFT (version 7.407) (Katoh and Standley, 2013). To evaluate the functional divergence between *A. thaliana* and *A. lyrata*, the nonsynonymous and synonymous substitutions in the 20,498 orthologs were counted. The K_A_/K_S_ ratio was calculated according to Yang and Nielsen’s method in the “yn00” program of PAML (version 4.8a) (Yang and Nielsen, 2000; Yang, 2007).

We compared the K_A_/K_S_ ratios of mQTGs, mQTGs with CNVs, and randomly selected genes (**Figure 1B** and **Supplementary Table S1**). The mQTGs were found to have significantly higher K_A_/K_S_ ratios than the randomly selected genes (Wilcoxon rank sum test: *P* < 0.001; **Figure 1B**), indicating that functional divergence was more commonly detected for mQTGs than for the other genes. Additionally, the K_A_/K_S_ ratios were higher for mQTGs with CNVs than for mQTGs and randomly selected genes (Wilcoxon rank sum test: *P* < 0.001; **Figure 1B**), suggesting that CNVs enhanced the functional divergence of mQTGs between *A*. *thaliana* and *A*. *lyrata*.

### Selection Pressure for CNVs in a Species Lineage

A strong positive selection decreases the nucleotide diversity around target sites throughout the genome (i.e., selective sweep). The mQTGs with CNVs are more frequently affected by a selective sweep than the other genes in *A*. *thaliana* accessions (Shirai et al., 2017). This suggests that CNVs contribute to local adaptations at the intra-species level of evolution. The results of the present study suggest that CNVs contribute to the functional divergence of mQTGs at the inter-species and intra-species levels. However, it remains unclear whether positive or relaxed selection pressure controls mQTGs with CNVs at the inter-species level of evolution.

In earlier investigations, determining the selection pressure generally involved comparisons between variations at the inter-species and intra-species levels of evolution (McDonald and Kreitman, 1991; Rand and Kann, 1996; Smith and Eyre-Walker, 2002; Stoletzki and Eyre-Walker, 2011). These studies compared the number of nonsynonymous mutations (P_n_), the number of synonymous mutations (P_s_), the number of nonsynonymous substitutions (D_n_), and the number of synonymous substitutions (D_s_). The neutrality index (NI; i.e., P_n/s_/D_n/s_) is one of the parameters for comparing the variations and inferring the selection pressure (Rand and Kann, 1996). The NI quantifies the direction and extent of the difference from neutrality in which P_n/s_ equals D_n/s_. That is, an NI of 1 means the intra-species and inter-species functional divergences are the same. Additionally, NI < 1 and NI > 1 reflect greater inter-species and intra-species functional divergences, respectively. Moreover, NI < 1 and NI > 1 represent the effects of positive and negative selection, respectively. We calculated the NI based on the variations of mQTGs with CNVs within *A*. *thaliana* accessions (intra-species) and between *A*. *thaliana* and *A*. *lyrata* (inter-species) among 20,214 genes. The P_n_ and P_s_ were estimated according to the SNPs of the 270 accessions (dN_SNP_/dS_SNP_ calculation). The D_n_ and D_s_ were estimated based on the substitutions of the orthologs between *A*. *thaliana* and *A*. *lyrata* (K_A_/K_S_ calculation).

We found that mQTGs and mQTGs with CNVs tend to have a lower NI than the randomly selected genes (Wilcoxon rank sum test: *P* < 0.05; **Figure 1C** and **Supplementary Table S1**). These results indicate that mQTGs and mQTGs with CNVs enhanced the inter-species functional divergence over the intra-species functional divergence. To address whether mQTGs with CNVs are associated with positive selection due to functional divergence, we examined the proportion of mQTGs with CNVs in positively selected genes and in other genes. We defined positively selected genes as a gene with NI < 1 and a significant difference between P_n/s_ and D_n/s_ (false discovery rate < 0.05 according to the chi-squared test; **Supplementary Table S1**). The proportion of mQTGs with CNVs (0.37% = 4/1,076) was significantly higher for positively selected genes than for the other genes (0.22% = 45/19,138) (chi-squared test: *P* = 2.42 × 10^−49^; **Supplementary Table S2**). These results imply that CNVs tend to be contained in the mQTGs related to the adaptive evolution of *A*. *thaliana*.

The NI reportedly leads to the incorrect determination of natural selection when there is an insufficient number of substitutions and mutations (Stoletzki and Eyre-Walker, 2011). Therefore, we validated the inferred selection pressure based on the direction of selection (DoS) (Stoletzki and Eyre-Walker, 2011). The DoS was defined as D_n_/(D_n_ + D_s_) − P_n_/(P_n_ + P_s_). Additionally, DoS > 0 and DoS < 0 represent the effect of positive and negative selection, respectively. We defined positively selected genes as genes with DoS > 0 and a significant difference between P_n/s_ and D_n/s_ (false discovery rate < 0.05 according to the chi-squared test; **Supplementary Table S1**). We examined the proportion of mQTGs with CNVs in positively selected genes and in other genes. Similar to the results of our analyses of NI, the proportion of mQTGs with CNVs (0.37% = 4/1,090) was significantly higher for positively selected genes than for the other genes (0.23% = 46/19,440) (chi-squared test: *P* = 6.12 × 10^−46^; **Supplementary Table S3**). Thus, the DoS analysis supported the NI results.

### Conclusion and Perspectives

**Figure 2:**
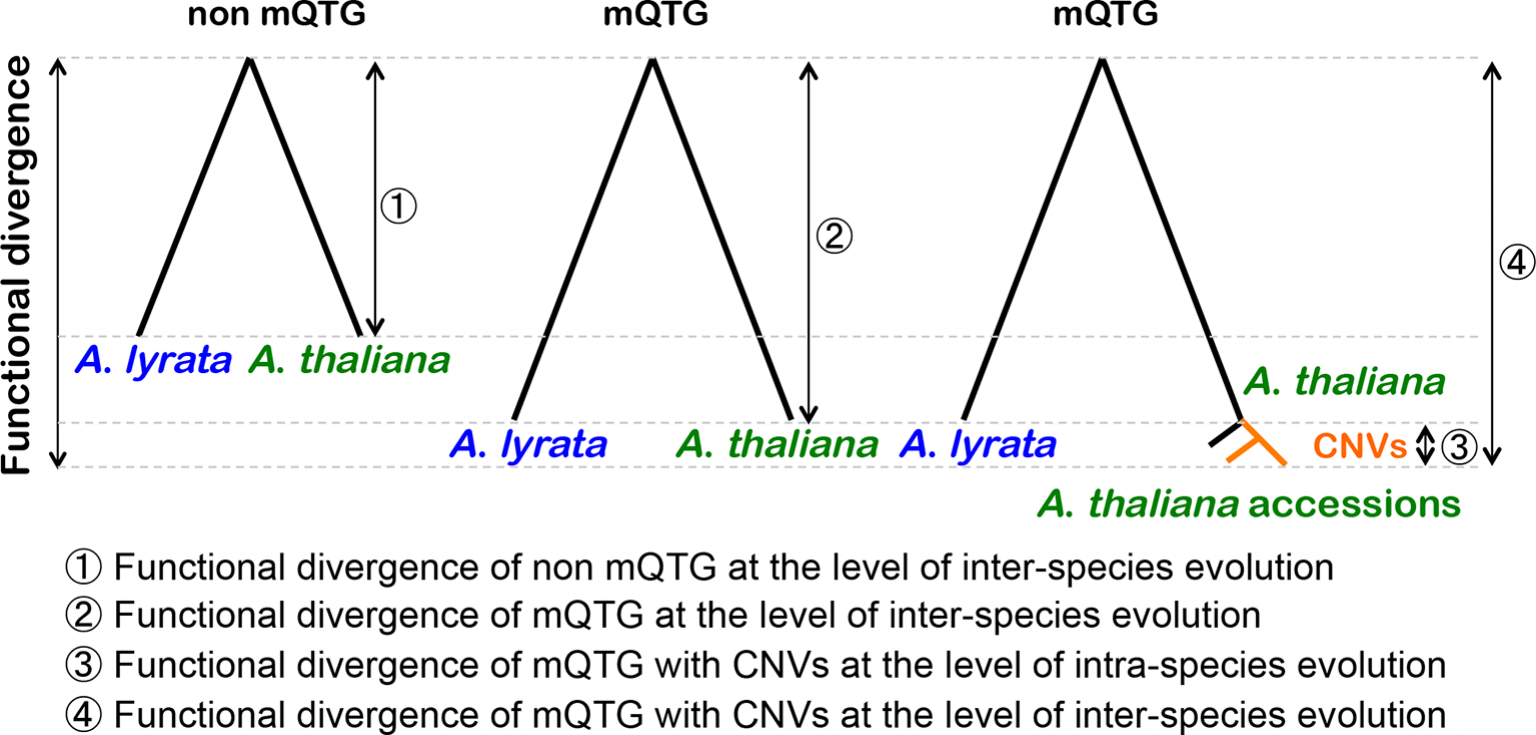
Contribution of CNVs to the functional divergence of mQTGs at the inter-species and intra-species levels. The functional divergence of the metabolic quantitative trait genes (mQTGs) is higher than that of the other genes (non-mQTGs) between *Arabidopsis lyrata* and *Arabidopsis thaliana* (inter-species level). In *A*. *thaliana*, mQTGs tend to have copy number variants (CNVs) because of gene duplications. After a gene duplication event, the duplicated copies accumulate nonsynonymous mutations. This causes the functional divergence of the mQTGs among *A*. *thaliana* accessions (intra-species level). Consequently, CNVs induce the functional divergence of mQTGs between *A*. *lyrata* and *A*. *thaliana*.

The current study examined the relationship between CNVs and the functional divergence of mQTGs at the inter-species and intra-species levels of evolution (**Figure 2**). Gene duplications induce nonsynonymous mutations *via* relaxed selection pressures. The CNVs derived from gene duplications seem to have accelerated nonsynonymous mutations. Thus, the mQTGs with CNVs have a high functional divergence at the intra-species level of evolution. Additionally, this intra-species functional divergence increases the inter-species functional divergence of the mQTGs. In fact, the functional divergence of mQTGs with CNVs tends to be high between *A*. *thaliana* and *A*. *lyrata*. Therefore, CNVs contribute to the functional divergence related to the diversity in specialized metabolites at the inter-species and intra-species levels. Consequently, CNVs tend to contribute to adaptations at the inter-species and intra-species levels. We propose that CNV is an important adaptive mechanism for generating diverse specialized metabolites in plants.

Our analyses are based on SNP calling with short-read sequencing. When SNPs are predicted in genes with CNVs based on the short reads, the SNPs are detected in the representative sequence of copied genes. The SNP detection over- or under-estimates the number of SNPs depending on the number of copied genes. In this study, we focused on only the rate of nonsynonymous or synonymous mutations. It is unlikely that the miscalling of SNPs between nonsynonymous and synonymous mutations is biased. Therefore, we believe that the effect of miscalling is limited for our analyses.

In the past 10 years, short-read sequencing has mainly been applied in investigations at the genome scale. Unfortunately, detecting structural variants is difficult based on short-read sequencing (van Dijk et al., 2018). Therefore, there have been relatively few studies on the CNVs in plants. However, third-generation sequencing platforms, such as Pacific Bioscience (PacBio), that can generate long reads (> 5 kb) have recently been applied for plant genomic research (Zhang et al., 2016; Fukushima et al., 2017; Lan et al., 2017; Baek et al., 2018; Edger et al., 2019). The long-read sequencing data may enable the accurate detection of structural variants (Jiao and Schneeberger, 2017; van Dijk et al., 2018). For example, structural variants were recently detected by PacBio in a tropical maize inbred line (Yang et al., 2019). If this experimental approach becomes more affordable, CNVs in plants may be more easily detected. Therefore, in the near future, it will be possible to verify conclusions in other plant species.

## Data Availability Statement

The datasets for this study are available in the 1001 Genomes (http://1001genomes.org), TAIR10 (https://www.arabidopsis.org), and Phytozome v12 (http://genome.jgi.doe.gov) databases.

## Author Contributions

KS analyzed the data and wrote the manuscript. KS and KH designed the data analysis method and revised and approved the manuscript.

## Funding

This work was supported by Grants-in-Aid for Scientific Research (25710017, 15H02433, 17H03727, 18KK0176, 18H02420, and 19H05348; to KH) as well as research grants from the Takeda Science Foundation (to KH), the Sumitomo Foundation (to KH), Kurume Research Park (to KH), and the Asahi Glass Foundation (to KH).

## Conflict of Interest

The authors declare that the research was conducted in the absence of any commercial or financial relationships that could be construed as a potential conflict of interest.

## Abbreviations

CNV, copy number variant; mQTG, metabolic quantitative trait gene; NI, neutrality index; PacBio, Pacific Bioscience; SNP, single nucleotide polymorphism.
