# Supplementary material for: Contribution of Functional Divergence Through Copy Number Variations to the Inter-Species and Intra-Species Diversity in Specialized Metabolites
Source: Front Plant Sci. 2019 Nov 26;10:1567. doi: 10.3389/fpls.2019.01567 (PMC6902010; doi:10.3389/fpls.2019.01567)
Supplement: Supplementary file 3 [file Presentation_1.pdf]

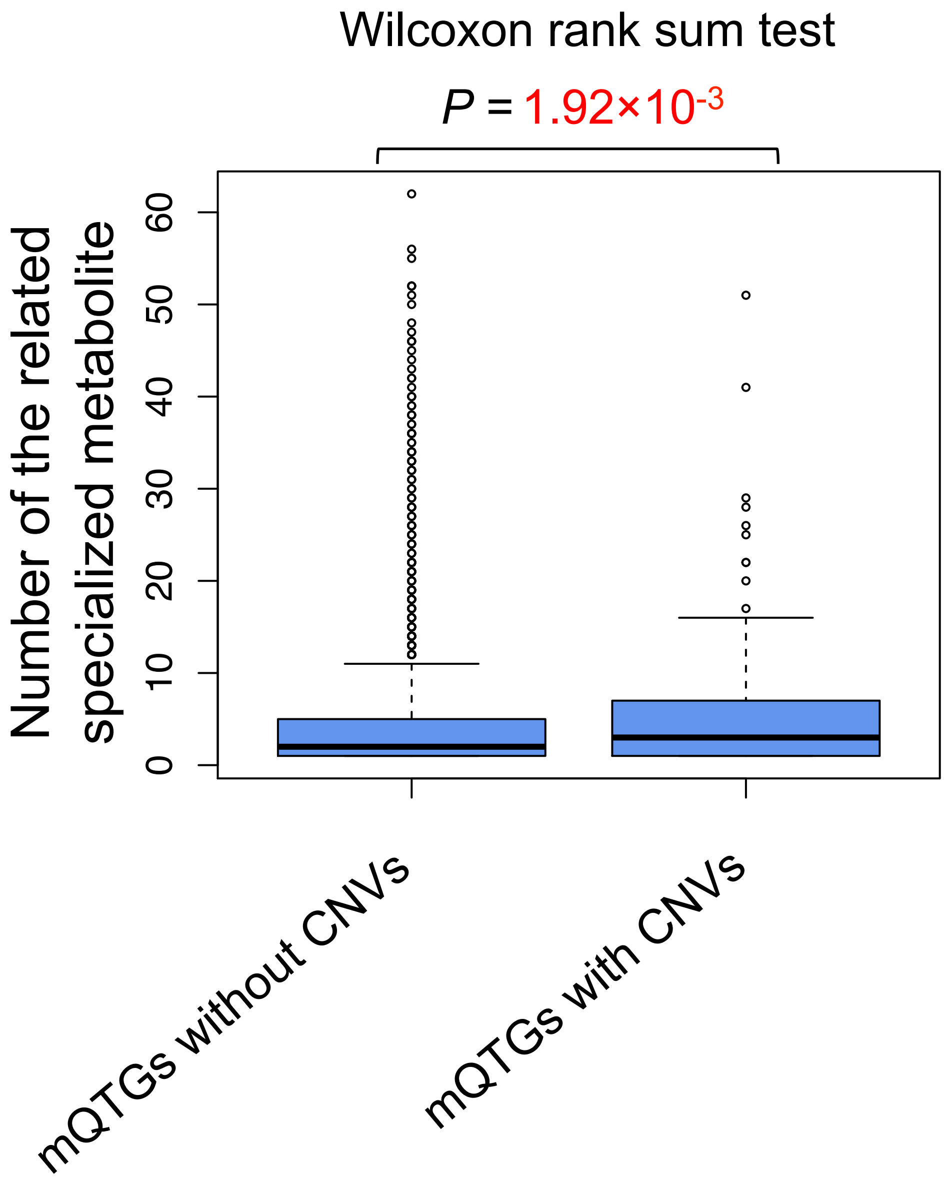

Supplementary Figure S1. Comparison of the number of the related specialized metabolites in Arabidopsis.

The box plots present the number of the related specialized metabolites of mQTGs in *Arabidopsis thaliana*. mQTGs without CNVs, metabolic quantitative trait genes without copy number variants. mQTGs with CNVs, metabolic quantitative trait genes with copy number variants. In each box plot, the box represents the 25%–75% range, the middle line represents the median, the dotted line represents the 1%–99% range, and the outer circles are outliers. *P* values were calculated by Wilcoxon rank sum test.

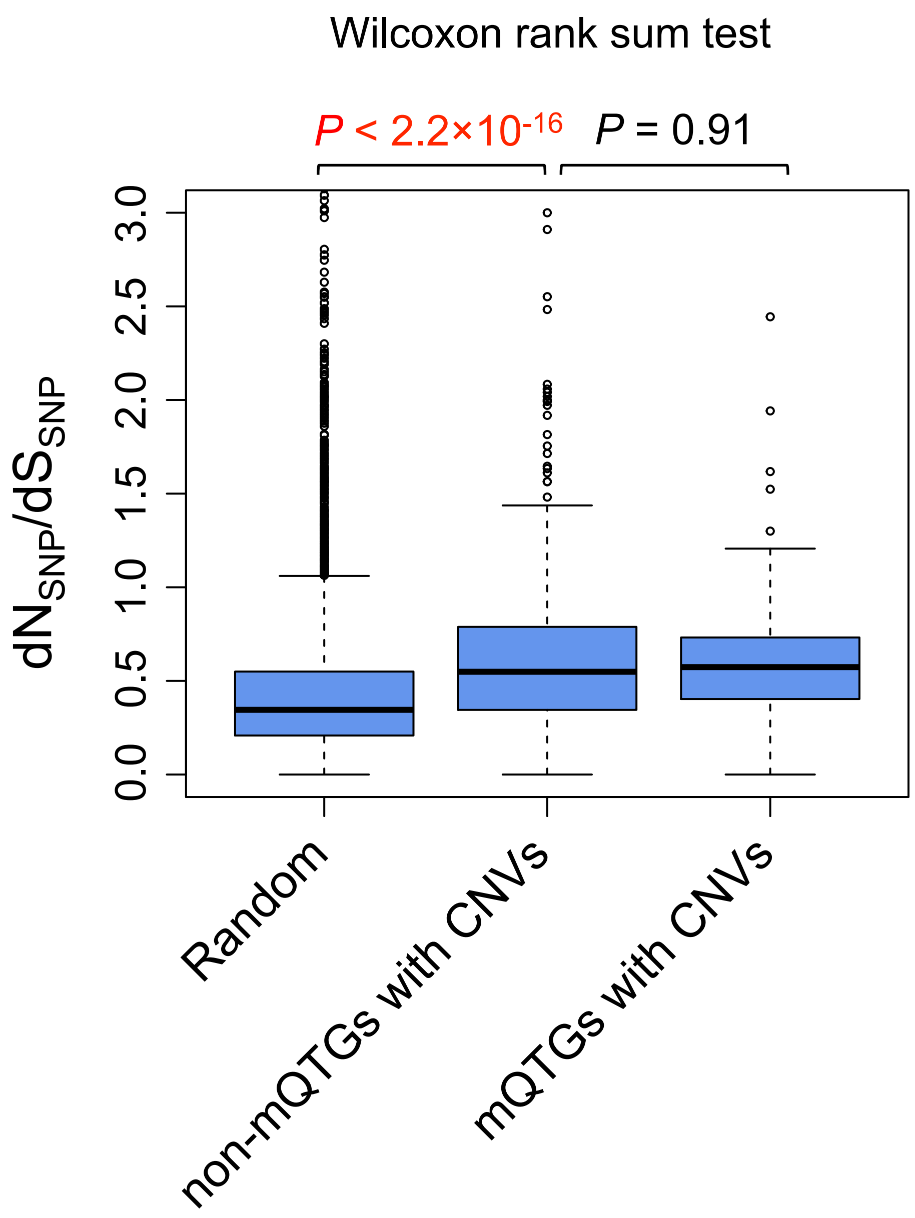

Supplementary Figure S2. Comparison of the functional divergence in Arabidopsis.

The box plots present the dN_{SNP}/dS_{SNP} ratios of *Arabidopsis thaliana* accessions. Random, 10,000 randomly selected genes; non-mQTGs with CNVs, non metabolic quantitative trait genes with copy number variants; mQTGs with CNVs, metabolic quantitative trait genes with copy number variants. In each box plot, the box represents the 25%–75% range, the middle line represents the median, the dotted line represents the 1%–99% range, and the outer circles are outliers. *P* values were calculated by Wilcoxon rank sum test.
